# A Smart Autonomous Time- and Frequency-Domain Analysis Current Sensor-Based Power Meter Prototype Developed over Fog-Cloud Analytics for Demand-Side Management

**DOI:** 10.3390/s19204443

**Published:** 2019-10-14

**Authors:** Yung-Yao Chen, Yu-Hsiu Lin

**Affiliations:** 1 Graduate Institute of Automation Technology, National Taipei University of Technology, Taipei 106, Taiwan; yungyaochen@mail.ntut.edu.tw; 2 Department of Electrical Engineering, Ming Chi University of Technology, New Taipei City 24301, Taiwan

**Keywords:** artificial intelligence, demand-side management, fog-cloud analytics, Industry 4.0, internet of things, machine learning, smart grid, smart homes

## Abstract

Electrical energy management, or demand-side management (DSM), in a smart grid is very important for electrical energy savings. With the high penetration rate of the Internet of Things (IoT) paradigm in modern society, IoT-oriented electrical energy management systems (EMSs) in DSM are capable of skillfully monitoring the energy consumption of electrical appliances. While many of today’s IoT devices used in EMSs take advantage of cloud analytics, IoT manufacturers and application developers are devoting themselves to novel IoT devices developed at the edge of the Internet. In this study, a smart autonomous time and frequency analysis current sensor-based power meter prototype, a novel IoT end device, in an edge analytics-based artificial intelligence (AI) across IoT (AIoT) architecture launched with cloud analytics is developed. The prototype has assembled hardware and software to be developed over fog-cloud analytics for DSM in a smart grid. Advanced AI well trained offline in cloud analytics is autonomously and automatically deployed onsite on the prototype as edge analytics at the edge of the Internet for online load identification in DSM. In this study, auto-labeling, or online load identification, of electrical appliances monitored by the developed prototype in the launched edge analytics-based AIoT architecture is experimentally demonstrated. As the proof-of-concept demonstration of the prototype shows, the methodology in this study is feasible and workable.

## 1. Introduction

Electricity is one of the most commonly used forms of energy in modern society. Consumer electrical energy demands are continuously increasing. During the last few decades rapid growth of Internet of Things (IoT) technologies has occurred. Modern IoT technologies have been developed and implemented for smart grids. A smart grid exploiting modern IoT technologies for a more efficient, reliable, and flexible traditional power grid upgraded in this revolution can be regarded as the key enabler that makes existing cities ready for tomorrow’s needs, including continuously increasing demands for electrical energy. A smart grid improves two-way communication between power utilities and consumers and opens new technical challenges, such as intelligent sensing and decision making. In a smart grid exploiting modern IoT technologies, to redefine the role of end users as power producers and the role of power utilities as service providers, consumers are “prosumers,” which allows them to generate, store, and sell their own electrical energy. Demand-side management (DSM) is very attractive and the most promising enabling technology of smart grids developed by power utilities and adopted to entice consumers to actively participate in demand response (DR) schemes [1,2,3,4]. DSM refers to initiatives [5] and technologies that encourage consumers to optimize their electrical energy consumption such that: (1) electrical energy requests from downstream sectors of a smart grid can be met; (2) the efficiency, reliability, and flexibility of the traditional power grid can be upgraded and enhanced; and (3) greenhouse gas emissions can be abated. Effective DSM alleviates such continuously increasing electrical energy demands. The benefits of participating in DSM are potentially twofold. First, consumers can reduce their electricity bills by adjusting their electrical energy consumption. Second, the power grid will adopt distributed energy resources, such as renewables and electric vehicles, which forces power utilities to rethink how the power grid can work intelligently and beneficially by shifting electrical energy consumption from peak hours to non-peak hours, in order to meet consumers’ electrical energy demands with flexible load shapes [6].

Smart energy management systems (EMSs) such as a home EMS, which have been considered as an integral part of successful DSM in a smart grid [7], can contribute to cutting electricity production costs by power utilities while still meeting electrical energy demands from downstream sectors of the smart grid. Many recent central EMSs [8,9,10,11,12] for DSM in a smart grid produce huge amounts of IoT data and transmit the data to resource-rich centralized data centers for data science analytics. However, networks may be congested, and centralized data centers and cloud analytics may be overloaded [13]. In addition, in cases where fast response with low communication overhead is required, user-centric IoT service-oriented EMS applications are network latency-sensitive and require real-time responsiveness and local interpretable and actionable data science analytics for rapid decision making (in case of immediate and adverse events) [14,15].

Centralized cloud analytics usually cannot remedy such drawbacks and fill such requirements [15]. Also, IoT and artificial intelligence (AI) (aka machine learning) have joined together to be envisioned as the next wave in the era of the IoT; IoT devices need informative AI models learned from IoT data in cloud analytics and used as edge analytics to provide real-time and onsite actionable data insights to customers [16,17]. Edge analytics and fog analytics, instead of cloud analytics, move cloud analytics close to IoT end devices to minimize network latency and enhance location awareness [18,19]. Not much attention has been paid to developing an EMS architecture over fog-cloud analytics for DSM in a smart grid. Thus, in this study, a smart autonomous time and frequency analysis current sensor-based power meter prototype, a novel IoT end device compared in [20,21,22,23], in an edge analytics-based AI across IoT (AIoT) architecture launched with an open and powerful cloud analytics platform is designed and implemented, which has assembled hardware and software to be developed over fog-cloud analytics for DSM in a smart grid. Fog-cloud analytics, which is considered for DSM in this study, concerned in a smart grid, and introduced conceptually in [24], concurrently leverages resources from cloud-centered data science analytics (advanced AI) to edge connectivity (advanced AI autonomously and automatically deployed onsite on the developed prototype via the Internet). AI well trained offline in cloud analytics and autonomously and automatically deployed as edge analytics on IoT end devices via the Internet was taken into account and developed for IoT applications in [15,21,22]. For a practical application of DSM in this study, auto-labeling, or load identification as nonintrusive load monitoring [8,10,25,26,27,28], of electrical appliances monitored by the developed prototype is experimentally investigated.

A summary of this study is as follows:

- A smart autonomous time- and frequency-domain analysis current sensor-based power meter prototype, which co-operates with cloud analytics (AI trained offline in cloud analytics) for collaborative learning and serves as edge analytics to identify electrical appliances onsite and online over fog-cloud analytics for DSM in a smart grid (well-trained AI autonomously and automatically deployed onsite on the prototype via the Internet (representational state transfer (REST) application programming interface (API))), is presented in this study. The prototype presented in our previous work [23] is upgraded in this study, which was based on time-domain feature extraction and was not autonomously and automatically collaborated with cloud analytics. The upgraded prototype in this study is a novel IoT end (edge) device, as compared with the work done in [20,21,22,23]. The prototype developed over fog-cloud analytics and presented in this study is experimentally examined. The prototype has assembled hardware and software to be developed.
- An open and powerful cloud analytics platform is configured and used as a data science analytics engine without developing custom web software for DSM to implement lightweight AI trained offline in cloud analytics and deployed, via the Internet (REST API), onsite on the prototype as edge analytics for online load identification in the presented fog-cloud analytics-based AIoT architecture.
- In AI, a model such as an artificial neural network (ANN) trying too hard to respond to noisy data in a training dataset should avoid overfitting. Regularization is one of the most important techniques used in AI to overcome overfitting. A radial basis function ANN (RBF-ANN) that takes a regularization risk function into account is employed in this study as lightweight AI. It has the low computational complexity of AI (lightweight AI) implemented in cloud analytics and deployed via the Internet (REST API) onsite on the presented prototype as edge analytics autonomously and automatically for online load identification in DSM. The RBF-ANN is a typical feed-forward ANN, which has the merits of (1) a simple network structure and (2) conspicuous fast and high learning and generalization performance.

This study is organized as follows: The developed prototype is presented in Section 2. Section 3 shows a proof-of-concept demonstration of the prototype developed over fog-cloud analytics for DSM, where the prototype is experimentally validated in a realistic household environment. Finally, this study is concluded in Section 4.

## 2. Methodology: Smart Autonomous Time and Frequency Analysis Current Sensor-Based Power Meter Prototype in an Edge Analytics-Based AIoT Architecture Launched with an Open Cloud Analytics Platform

**Figure 1:**
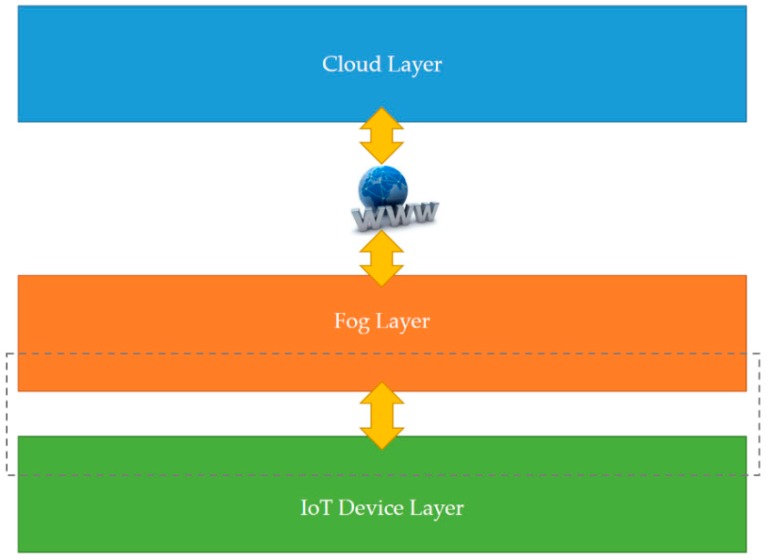
Generic three-tier architecture of fog-cloud analytics. IoT = Internet of Things.

**Figure 2:**
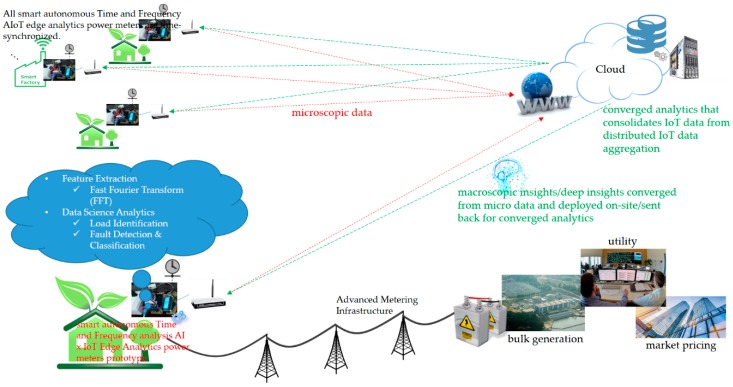
Conceptual vision of the smart autonomous power meter prototype considering time- and frequency-domain analysis (spectral analysis) and advanced artificial intelligence (AI) in a future smart sensing infrastructure based on fog-cloud analytics for demand-side management (DSM) in a smart grid.

This section presents the developed smart autonomous time and frequency analysis current sensor-based power meter prototype in an edge analytics-based AIoT architecture launched with an open and powerful cloud analytics platform, which is designed and implemented over fog-cloud analytics for DSM. Fog-cloud analytics is crucial for user-centric IoT applications in DSM in a smart grid, which is network latency-sensitive and requires real-time and onsite data science analytics for immediate decision making. Figure 1 depicts a generic three-tier architecture of fog-cloud analytics consisting of an IoT device layer, a fog layer, and a cloud layer [29,30]. In such a generic architecture, IoT data sensed and gathered in an IoT device layer are exchanged with a fog layer that allows for communication of IoT end edge devices, including edge gateways (fog devices can be gateways [31]), with a powerful cloud analytics platform. An IoT device layer is the basis of a generic architecture of fog-cloud analytics. IoT end edge devices are the main entities of an IoT device layer, and they can be deployed over a wide area of interest for user-centric IoT applications. A configured fog layer is used as a technical bridge between an IoT device layer and a cloud layer to process gathered IoT data through data science analytics at IoT data sources and exchange processed IoT data via IoT communication technology [32]. A fog layer is made of edge gateways that respond rapidly to IoT end edge devices in an IoT device layer, and IoT end edge devices, including edge gateways, avoid network latency over the Internet because of their proximity to them. A cloud layer, which can be distributed in cloud storage and user-centric IoT applications, is responsible for storing gathered and processed IoT data and providing collaborative user-centric IoT services to customers. In a generic architecture of fog-cloud analytics, IoT end edge devices, including edge gateways, in a fog layer gather processed IoT data from IoT end edge devices in an IoT device layer and pass them to a cloud layer. Mukherjee et al. in [33] conducted a comprehensive survey relating to fog-cloud analytics-based IoT architectures; interested researchers can refer to the survey article. This study develops an AIoT architecture based on fog-cloud analytics for DSM in a smart grid, which is similar to the generic architecture depicted in Figure 1. Figure 2 shows a conceptual vision of the developed smart autonomous power meter prototype as a new edge computing device [20,21,22,23] considering time and frequency analysis and advanced AI in a future smart sensing infrastructure based on fog-cloud analytics for DSM in a smart grid [23]. Future advances, (1) user-centric IoT service-oriented single and/or multiple sensing modalities and applications and (2) fault detection and classification in industry 4.0 (smart factories), for DSM in a smart grid can be built upon. As cloud analytics applied to gathered IoT data that need to be processed for real-time and onsite actionable data insights at IoT data sources is based on network connectivity that is not always available or is limited, edge (fog) analytics is a promising technique dedicated to data science analytics enabled directly at the edge of the network. In this sense, edge analytics extends cloud analytics and covers its shortage.

Fog-cloud analytics serves as converged analytics that consolidates IoT data from distributed IoT data aggregation. Based on fog-cloud analytics developed for user-centric IoT applications in smart homes, manufacturers, and cities considering DSM in a smart grid, as shown in Figure 2, the developed prototype, which is installed in multiple fields of interest in a smart grid, time-synchronized, and autonomously and automatically deployed onsite as edge analytics with advanced AI (trained offline in cloud analytics and ported on the prototype via the Internet), is able to perform converged analytics onsite and online. Converged analytics consolidates IoT data from distributed IoT data aggregation. The smart autonomous power meter prototype presented in this section and developed over fog-cloud analytics for DSM in this study is a preliminary design toward such a scenario as a next-generation AMI (smart sensing) infrastructure expected for smart homes, manufacturers, and cities.

### 2.1. Architectural Design of an Edge Analytics-Based AIoT Considering the Developed Smart Autonomous Time and Frequency Analysis Current Sensor-Based Power Meter Prototype with Open Cloud Analytics for DSM in a Smart Grid

**Figure 3:**
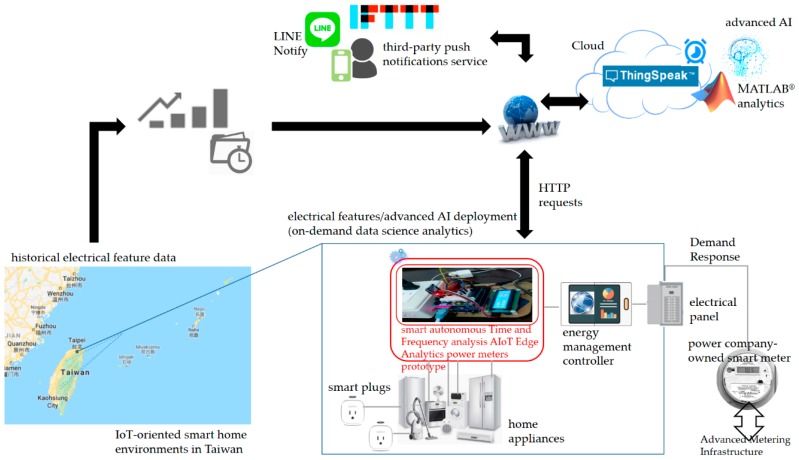
Launched edge analytics-based artificial intelligence across Internet of Things (AIoT) with open and powerful cloud analytics configured to collaborate with the developed smart autonomous time and frequency analysis current sensor-based power meter prototype as edge analytics over fog-cloud analytics for DSM in a smart grid in this study.

Figure 3 illustrates the architecture of the prototype designed and implemented in this study, which is a version of a single-home demonstration of the generic three-tier fog-cloud analytics architecture depicted in Figure 1. In Figure 3, the fog-cloud analytics architecture is implemented in a home in Taiwan. The residential environment is equipped with an energy management controller that manages home appliances in response to DR signals and is connected via an advanced metering infrastructure to a smart grid that comes up with DR programs in which a power company-owned smart meter instead of a traditional wattmeter is installed and used to transmit electrical energy consumption data records to and receive DR signals from a power utility for DSM. One of the most important functionalities of a smart grid is self-decision-making ability [34]. To enable this functionality, DR, a viable approach to motivate consumers toward shifting their electrical energy demand during peak load periods [35], emerges. As electrical appliances monitored in the residential environment in Figure 3 are identified and reacted with DR signals, the developed prototype in this study is used to identify them based on time- and frequency-domain analysis.

The developed smart autonomous power meter prototype in the residential environment in a smart grid is time-synchronized and autonomously and automatically deployed onsite as edge analytics, which is designed and implemented to interact with an open and powerful cloud analytics platform configured for advanced AI trained offline in cloud analytics and ported on the prototype via the Internet. The cloud analytics platform configured to collaborate with the prototype over fog-cloud analytics is based on ThingSpeak™ [36] with MATLAB^®^ analytics, which can run MATLAB^®^ codes on demand in the cloud. In the architecture, push notifications service is also developed. The push notifications service provided in the architecture in Figure 3, If-This-Then-That (IFTTT) [37], with Webhooks [38] publishing a new trigger received or action alerted is conducted, configured, and used for LINE-Notify mobile devices. The developed prototype is presented in Section 2.1.1. The open and powerful cloud analytics platform based on ThingSpeak™ with MATLAB^®^ analytics is presented in Section 2.1.2, where advanced AI implemented in MATLAB^®^ programming language, trained offline in cloud analytics and ported as edge analytics on the developed prototype via the Internet for onsite and online load identification in DSM, is mathematically described in Section 2.2.

#### 2.1.1. Hardware: Smart Autonomous Time and Frequency Analysis Current Sensor-Based Power Meter Prototype as Edge Analytics

**Figure 4:**
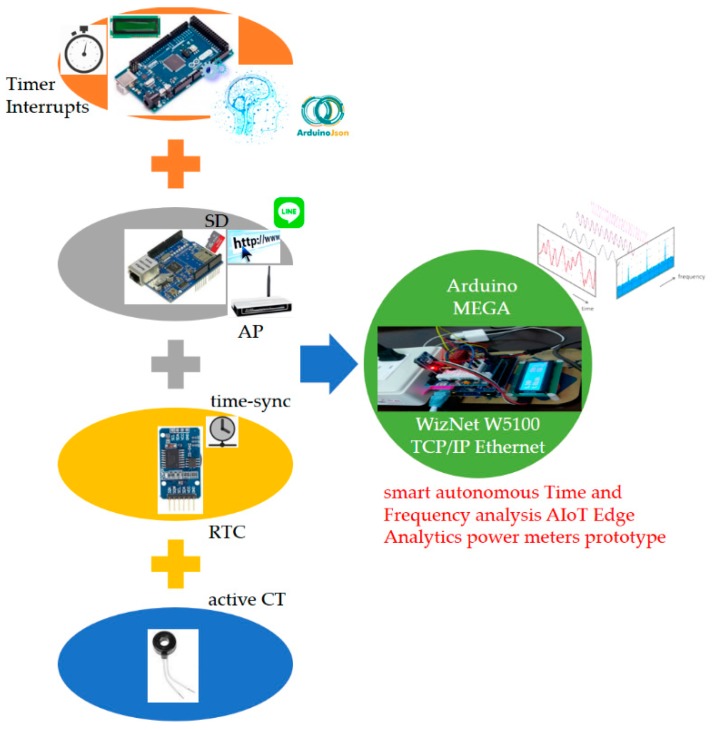
Generic three-tier architecture of fog-cloud analytics.

The smart autonomous time and frequency analysis current sensor-based power meter prototype designed and implemented as edge analytics with push notifications service in Figure 3 is depicted in Figure 4. The core entity of the prototype in Figure 4 is based on an Arduino^®^ micro-controller unit (MCU) board. More specifically, Arduino MEGA 2560 [21,23,39] is chosen and designed with hardware and software implementation in this study. Arduino^®^, an open-source and inexpensive electronics prototyping MCU based on flexible and easy-to-use hardware and software, has become very popular among artists, designers, hobbyists, and professionals. It is an excellent IoT tool to quickly test and deploy IoT ideas and applications. Arduino^®^ is programed in Arduino^®^ language, which is based on C/C++ programming language and comes with a user-friendly integrated development environment (IDE) [40]. The Arduino MEGA 2560 MCU used in this study provides sufficient analog pins for possible future uses in smart homes.

The general specifications of the Arduino MEGA 2560 MCU are listed in Table 1. It is an MCU board based on the Atmel^®^ 8-bit ATmega2560. It has 54 digital input/output (I/O) pins (of which 15 can be used for pulse width modulation (PWM)), 16 analog inputs, four universal asynchronous receivers/transmitters (UARTs; hardware serial ports), a larger memory space for a coded Arduino sketch, a 16 MHz crystal oscillator, a universal serial bus (USB) connection, a power jack, an in-circuit serial programming (ICSP) header, and a reset button. In an Arduino^®^ MCU board, the flash memory, program space, is where an Arduino sketch coded is stored. The static random access memory (SRAM) is where the coded Arduino sketch creates and manipulates variables when the board runs. The electrically erasable programmable read-only memory (EEPROM) is the memory space that programmers can use to store long-term data. 

The Arduino MEGA 2560 MCU is compatible with most Arduino^®^ shields, such as a WIZNet W5100 hardwired Transmission Control Protocol/Internet Protocol (TCP/IP) embedded Ethernet shield [41], produced for Arduino UNO, and former microcontroller boards such as Arduino Duemilanove. A WIZNet W5100 hardwired TCP/IP embedded Ethernet shield is used in this study for the developed prototype (Figure 4). The designed and implemented prototype has the following modules:

(1) A current transducer (CT) coil, which is clipped on a live electrical wire and used to acquire current signals. Acquired current signals reflect electrical appliances monitored by the prototype, analyzed through time- and frequency-domain analysis, and identified by advanced AI. For a CT connected to an Arduino^®^ MCU, the output signal from the CT needs to be conditioned so it meets the input requirements, positive voltage between 0 V and the ADC reference voltage (AREF; 5 V on 5 V MCU boards or 3.3 V on 3.3 V boards), of the Arduino^®^ MCU’s analog inputs.
(2) A WIZNet W5100 hardwired TCP/IP embedded Ethernet shield, which is used to support Internet connectivity where data processed through time and frequency analysis are transmitted to the open and powerful cloud analytics platform in Figure 2 and Figure 3 for advanced AI, and where advanced AI well trained offline in cloud analytics is retrieved from the cloud analytics platform and autonomously and automatically deployed as edge analytics on the Arduino MEGA 2560 MCU in a regular periodic manner.
(3) A real-time clock (RTC) chip, which is used to keep track of current time, timestamps, via network time protocol (NTP; the developed prototype designed and implemented to interact with the cloud analytics platform in Figure 2 and Figure 3 for advanced AI trained offline in cloud analytics and ported as edge analytics on the prototype via the Internet is time-synchronized).
(4) A micro SD card, which is used to store data (the SD library built on ‘sdfatlib’ by Greiman allows for reading data from and writing data to the SD card, and data gathered are reliably replicated on the cloud analytics platform).
(5) Advanced AI, which is ported from the cloud analytics platform to the developed prototype via the Internet and used as edge analytics to identify electrical home appliances (represented as electrical features) based on time- and frequency-domain analysis onsite and online.

Wireless communication technology such as Bluetooth [22], ZigBee [42], Wi-Fi [43,44], and 2G General Packet Radio Services (GPRS) [45] can be conducted and developed in the architecture in Figure 3. In this sense, the WIZNet W5100 hardwired TCP/IP embedded Ethernet shield mounted on the Arduino MEGA 2560 MCU, the core entity of the designed and implemented smart prototype shown in Figure 4 can be replaced with an ESP8266 ESP-01 system-on-a-chip (SoC) Wi-Fi microchip or an ESP8266 ESP-12E Wi-Fi SoC NodeMCU [46] where advanced AI well trained offline in cloud analytics can be autonomously and automatically deployed onsite on the NodeMCU as edge analytics via the over-the-air programming.

The process of data science analytics comprising feature extraction and load identification by the developed prototype considering advanced AI for DSM is shown in Figure 2. Below, feature extraction is described. Advanced AI well trained offline in cloud analytics and autonomously and automatically deployed onsite on the developed prototype as edge analytics is mathematically described in Section 2.2.

To enable feature extraction for load identification in DSM in this study, real power (P) [46,47], turn-on transient power [46], and current harmonics extracted from current signals conditioned and acquired by the CT are the electrical features identified by advanced AI. The turn-on transient power is defined as the real power, which is consumed by an electrical appliance plugged into the developed prototype and monitored, that transient power is captured and extracted when the appliance is turned on (the appliance turned on will settle down). For current harmonics, the conditioned and acquired current signals in the time domain are transformed by fast Fourier transform (FFT) [48,49,50,51,52,53] into the frequency domain.

The FFT is an algorithm that performs a discrete Fourier transform; it is a mathematical tool that allows signals conditioned and acquired in the time domain to be displayed in the frequency domain, and it is widely used in signal processing. The FFT performed for feature extraction for load identification in DSM in this study was implemented in Arduino^®^ language [52] and run on the Arduino MEGA 2560 MCU. In the Arduino^®^ IDE, programmers only need to program two functions to make an executable Arduino^®^ sketch: *setup()* and *loop()*. The *loop()* function is not ideal for high-speed data acquisition, as it runs continuously without using a timer [54]. Instead, interrupts are used to allow predictable timing for high-speed data acquisition. In this study, interrupts triggered for high-speed data acquisition were implemented by the FlexiTimer2 library [55]. FlexiTimer2, which is based on MsTimer2 [56] by Javier Valencia, offers more time-spatial flexibility than MsTimer2, since it has a configurable submillisecond timer resolution comprising the 1 millisecond timer resolution on timer2 for an Arduino^®^ MCU’s interrupts. In an Arduino^®^ MCU, events that trigger interrupts are internal timer overflows. Each time there is a timer overflow, a callback routine is called and executed. In our case, the callback routine is a function in which raw current data are read through *analogRead()* from a specified analog pin of the MCU circuited with a CT (Figure 4). The MCU contains a multichannel and 10-bit analog-to-digital converter (ADC), which maps input voltages between 0 and 5 V into integer values ranging from 0 to 1023. *analogRead()* reads mapped integer values from the specified analog pin of the MCU, and they are shifted by their mean value, computed with a voltage step of ~4.88 mV, and then transformed by FFT to feature extraction for load identification in DSM.

To enable load identification for DSM in this study, advanced AI trained offline in cloud analytics (i.e., applied on electrical features extracted through feature extraction for onsite and online load identification and transmitted to the cloud analytics platform for advanced AI in the architecture shown in Figure 2 and Figure 3) is autonomously/automatically deployed onsite on the developed smart autonomous power meter prototype as edge analytics and used to identify electrical appliances. Across the architecture over fog-cloud analytics, load identification for DSM in this study is performed onsite and online.

The cloud analytics platform that collects data transmitted from the developed smart autonomous power meter prototype as edge analytics for advanced AI and runs advanced AI trained offline in cloud analytics for DSM is described in Section 2.1.2.

#### 2.1.2. Software: Open and Powerful Cloud Analytics Platform Based on ThingSpeak™ with MATLAB^®^ Analytics

**Figure 5:**
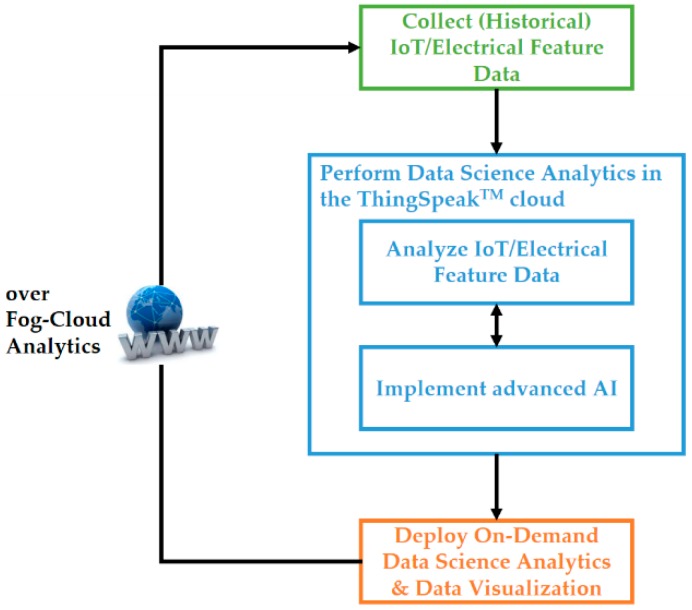
Workflow of the open and powerful cloud analytics platform, ThingSpeak™ with MATLAB^®^ analytics, configured over fog-cloud analytics for DSM in this study.

The open and powerful cloud analytics platform that collects data transmitted from the developed smart autonomous power meter prototype as edge analytics for advanced AI and runs advanced AI trained offline in cloud analytics for DSM is described here. As shown in Figure 3, the cloud analytics platform as a data science analytics engine without developing custom web software for DSM is based on ThingSpeak™ [36] with MATLAB^®^ analytics. ThingSpeak™ is a free, open, and powerful IoT platform that can run MATLAB^®^ codes on demand in the cloud. Also, it offers an easy way to collect IoT data from things for IoT application developers, analyze data, and visualize data processed by MATLAB^®^, where application developers can act on their collected and processed IoT data [36]. With MATLAB^®^ from MathWorks^®^, application developers have access to powerful toolboxes such as the Signal Processing Toolbox™, Statistics and Machine Learning Toolbox™, and Neural Network Toolbox™ for their analyzed and visualized IoT data. Figure 5 shows the workflow of the cloud analytics platform, ThingSpeak™ with MATLAB^®^ analytics, configured over fog-cloud analytics for advanced AI implemented for DSM in this study, which is completed in the following three steps:

(1) Collect (historical) IoT data (electrical feature data). Electrical features are extracted from appliances and learned by advanced AI in cloud analytics for DSM, such as load identification, fault detection and classification, and prognostics and health management. Advanced AI well trained in cloud analytics will be autonomously and automatically deployed on, or ported on, onsite the developed smart autonomous power meter prototype via the Internet for on-demand data science analytics and data visualization.
(2) Perform data science analytics in the ThingSpeak™ cloud.
  (2.1) Analyze collected IoT data (electrical feature data), which are statistically inspected and cleaned to remove noise, outliers, missing or erroneous values, and anomalies.
  (2.2) Implement advanced AI applied on analyzed data for interpretable and actionable insights (code advanced AI in a MATLAB® script), publish the coded script in the ThingSpeak™ cloud, and then schedule the published script to regularly/periodically execute the implemented advanced AI for DSM such as load identification, fault detection and classification, and prognostics and health management.
(3) Deploy on-demand data science analytics over fog-cloud analytics for the implemented advanced AI onsite on the developed prototype at the edge of the Internet. Over fog-cloud analytics, the prototype communicates with the ThingSpeak™ cloud for model updates and transmits analyzed and classified data to the ThingSpeak™ cloud for model scheduling and updating.

Advanced AI involved in the fog-cloud analytics architecture in this study is described in Section 2.2.

### 2.2. Advanced AI: K-Means Clustering Combined with RBF-ANN Trained Offline in Cloud Analytics and Autonomously and Automatically Deployed Onsite on the Developed Smart Autonomous Power Meter Prototype as Edge Analytics via the Internet

**Figure 6:**
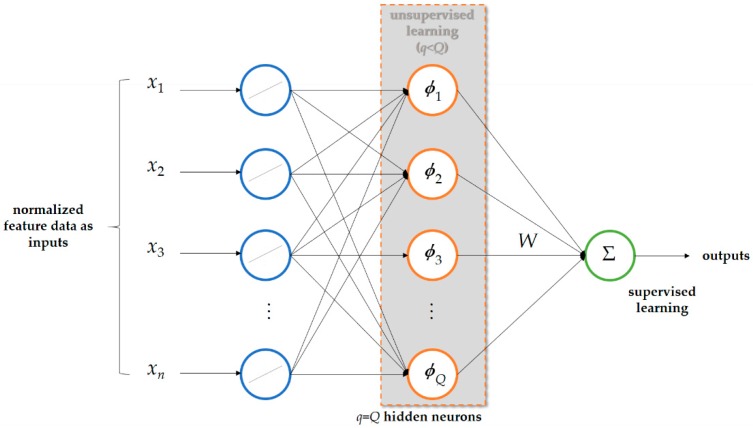
Standard radial basis function artificial neural network (RBF-ANN), lightweight AI.

A biologically inspired RBF-ANN [57] facilitated by k-means clustering [58,59,60] is developed and used as advanced AI trained offline in cloud analytics and autonomously and automatically deployed onsite on the developed smart autonomous power meter prototype as edge analytics over fog-cloud analytics for DSM. The RBF-ANN [57], a rather simple three-layer ANN, used in this study consists of one input layer, one hidden layer comprising artificial neurons with radial basis functions taking on the role of nonlinear activation functions, and one output layer, as shown in Figure 6. The RBF-ANN has conspicuous fast and high learning and generalization performance. In this study, k-means clustering, also known as hard c-means clustering or Lloyd’s algorithm [58], is used to characterize the standard RBF-ANN in Figure 6. K-means clustering is a data-partitioning-based clustering algorithm that assigns data points into k clusters (centroids) across a number of variables through an iterative process.

Let $ℑ={\left\{X_{k}{,}^{}d_{k}\right\}}_{k=1}^{Q}$ be a training dataset containing Q input–output pairs, where $X_{k}\in R^{n}$ and $d_{k}\in R$. The goal of training the RBF-ANN is to search for map *f* that takes each input $X_{k}$ ($k=1{,}^{}\ldots {,}^{}Q$) and then maps exactly it onto its desired output $d_{k}$: $f(X_{k})=d_{k}$.

With the purpose of mapping $f(X_{k})=d_{k}$ to be learned through the training process of the RBF-ANN, the RBF-ANN assumes a set of exactly *q* nonlinear basis functions $\phi (\|X_{k}-\mu_{i}\|)$ whose argument involves a Euclidean distance metric ${\|X}_{k}-\mu_{i}\|$. The Euclidean distance metric measures the distance between the *k*th input $X_{k}$ and the *i*th center (cluster mean) $\mu_{i}$, where *i* = 1, 2, …, *q* (= c cluster means found by k-means, or hard c-means, clustering) and $\mu_{i}\in R^{n}$. In this study, the radially symmetric Gaussian-style basis function is used.

Map *f* is then generated by taking a weighted linear superposition of *q* nonlinear basis functions, as given in Equation (1):

(1) $$f(X)=\sum_{i=1}^{q}w_{i}\phi ({\|X}_{k}-\mu_{i}\|)$$

where *w_i_* is the *i*th weight coefficient.

Map *f* is solved in a least squares sense, where the familiar squared error function that computes the squared error summed over all *Q* data points in the training dataset is introduced in Equation (2):

(2) $$\varepsilon =\frac{1}{2}\sum_{k=1}^{Q}[d_{k}-\sum_{i=1}^{q}w_{i}\phi ({\|X}_{k}-\mu_{i}\|){]}^{2}$$

To obtain the optimal weight coefficients in a least squares sense, we differentiate Equation (2) with respect to $w_{i}$ and set it equal to zero, as shown in Equation (3):

(3) $$\sum_{k=1}^{Q}\phi_{ki}(\sum_{l=1}^{q}w_{l}\phi_{kl})=\sum_{k=1}^{Q}d_{k}\phi_{ki}$$

By the following matrix definitions: 

$\Phi =\left[\begin{array}{ccc} \phi_{11} & \ldots & \phi_{1q}\\ \phi_{21} & \ldots & \phi_{2q}\\ \vdots & \ddots & \vdots \\ \phi_{Q1} & \ldots & \phi_{Qq} \end{array}\right]$, D = ${\left[d_{1}{,}^{}\ldots {,}^{}d_{Q}\right]}^{T}$, and W = ${\left[w_{1}{,}^{}\ldots {,}^{}w_{q}\right]}^{T}$, Equation (3) is recast into a matrix form, as shown in Equation (4):

(4) $$(\Phi^{T}\Phi )W=\Phi^{T}D$$

Finally, the weight vector *W* in Equation. (4) is solved by a singular value decomposition (SVD) technique [57], which is used to train the RBF-ANN once the specification of the center and spread parameters of each Gaussian basis function has been completed through the k-means clustering, as given in Equation (5):

(5) $$W={\left(\Phi^{T}\Phi \right)}^{-1}\Phi^{T}D=\Phi^{*}D$$

where $\Phi^{*}$, a *q*-by-*Q* matrix, is the pseudo-inverse [57,61].

From the regularization theory, where the standard error minimization problem is replaced with minimization of a regularization risk functional [57] applied to Equation (2) with a smoothness functional, Equation (5) becomes Equation (6):

(6) $$W={\left(G^{T}G+\lambda \tilde{G}\right)}^{-1}G^{T}D=G^{*}D$$

where **G** denotes a Green’s function (multivariate Gaussian is a Green’s function) [57]: $G=\left[\begin{array}{ccc} G({\|X}_{1}-\mu_{1}\|) & \ldots & G({\|X}_{1}-\mu_{q}\|)\\ G({\|X}_{2}-\mu_{1}\|) & \ldots & G({\|X}_{2}-\mu_{q}\|)\\ \vdots & \ddots & \vdots \\ G({\|X}_{Q}-\mu_{1}\|) & \ldots & G({\|X}_{Q}-\mu_{q}\|) \end{array}\right]$; $\tilde{G}=\left[\begin{array}{ccc} G({\|\mu }_{1}-\mu_{1}\|) & \ldots & G({\|\mu }_{1}-\mu_{q}\|)\\ G({\|\mu }_{2}-\mu_{1}\|) & \ldots & G({\|\mu }_{2}-\mu_{q}\|)\\ \vdots & \ddots & \vdots \\ G({\|\mu }_{q}-\mu_{1}\|) & \ldots & G({\|\mu }_{q}-\mu_{q}\|) \end{array}\right]$; and λ is a regularization parameter that controls the trade-off between the closeness to the fitted data and the smoothness of the regularized Tikhonov functional [57] (the Tikhonov functional, also called a stabilizer, forces the approximation to become as smooth as possible). The pseudo-inverse $G^{*}$ in Equation (6) trends to $\Phi^{*}$ in Equation (5) as λ→0 (there is no smoothness constraint; *W* is completely determined by the training dataset ℑ).

In this study, the radially symmetric Gaussian basis functions, $\phi (\|X_{k}-\mu_{i}\|)=\exp(-\frac{\|X_{k}-{\mu_{i}\|}^{2}}{2\sigma^{2}})$, involving the distributed center and spread parameters partially overlapped and heuristically determined by k-means clustering are adopted for the RBF-ANN in Equation (1) (Figure 6). Assuming that the Gaussian basis functions are centered at ${\left\{\mu_{i}\right\}}_{i=1}^{q}$ as cluster means by k-means clustering, we define the maximum distance α between any of the chosen center parameters as $\alpha =\underset{1\leq i,j\leq q}{\max}(\|\mu_{i}-\mu_{j}\|)$. Then, the spread parameters σ of the centered Gaussian basis functions are computed by Equation (7) [57]:

(7) $$\sigma =\frac{\alpha }{\sqrt{2q}}$$

Overall, Equation (6) admits the RBF-ANN shown in Figure 6. In summary, the standard RBF-ANN of Equation (1) characterized through k-means clustering, trained by Equation (6), and developed in this study is involved in the following two stages and is outlined in Algorithm 1:

(1) Stage 1. Apply k-means clustering to the onsite collected training data points, to heuristically determine the center and spread parameters of each Gaussian basis function of Equation (1). The Gaussian-type basis function’s centers are spanned, as computed by Equation (7). K-means clustering, which is used in this stage to characterize the RBF-ANN of Equation (1) trained in the following stage, is summarized below:
  *Step 1*. Suppose an onsite collected training dataset $ℑ={\left\{X_{k}{,}^{}d_{k}\right\}}_{k=1}^{Q}$ with $X_{k}\in R^{n}$ is given. Fix *c* (k-means or hard c-means clustering), where 2 ≤ *c* < *Q*. Initialize *U*^(0)^ ∈ *M_c_*, where *M_c_* = {*U* ∈ *V_cn_* | *u_ik_* ∈ {0, 1}, 1 ≤ *i* ≤ *c*, 1 ≤ *k* ≤ *Q*; $\sum_{i=1}^{c}u_{ik}=1,\forall k\in \{1,2,\ldots ,Q\}$; $0<\sum_{k=1}^{Q}u_{ik}<Q,\forall i\in \{1,2,\ldots ,c\}$ are true}; *V_cn_* is the set of real *c* × *Q* matrices *U* = [*u_ik_*] (hard c-partition space for ℑ).
  *Step 2*. At iteration *l*, where *l* = 0, 1, 2, …, compute the c-mean centers using Equation (8). In Equation (8), [*u_ik_*^(*l*)^] = *U*^(*l*)^ and *i* = 1, 2, … *c*:
    (8) $$V_{i}^{\left(l\right)}=\frac{\sum_{k=1}^{Q}X_{k}u_{ik}{}^{\left(l\right)}}{\sum_{k=1}^{Q}u_{ik}{}^{\left(l\right)}}$$
  *Step 3*. Update *U*^(*l*)^ to *U*^(*l*+1)^ = [*u_ik_*^(*l*+1)^] using Equation (9), where data points with short distances among them should be grouped together:
    (9) $$u_{ik}{}^{\left(l+1\right)}=\{\begin{array}{c} 1\;,\;\\ 0\;,\; \end{array}\begin{array}{c} \left||X_{k}-V_{i}{}^{\left(l\right)}||=\min_{1\leq j\leq c}(||X_{k}-V_{j}{}^{\left(l\right)}||\right)\\ \;otherwise \end{array}$$
  *Step 4.* Compare *U*^(*l*)^ with *U*^(*l*+1)^: if ||*U*^(*l*+1)^ − *U*^(*l*)^|| < *ε* for a prespecified small tolerance, stop; otherwise, set *l* = *l* + 1 and go to Step 2. The cluster centers found by Equation (8) through k-means clustering are used to heuristically determine the center and spread parameters of each Gaussian basis functions of Equation. (1). Data points with short distances among them are clustered together by k-means clustering and learned by an exponential of a distance measure between the data points and their cluster center that characterizes the Gaussian basis function at a hidden neuron in the hidden layer of the RBF-ANN in Figure 6.
(2) Stage 2. Use the SVD technique applied to Equation (6) with the training data points collected onsite to train the standard RBF-ANN of Equation (1) characterized in Stage 1. Once the k-means clustering combined with RBF-ANN has been well trained in the ThingSpeak^TM^ cloud, it is autonomously and automatically deployed onsite on (ported on) the developed smart autonomous power meter prototype as edge analytics via the Internet. Then, the developed prototype is used to perform online load identification for DSM.

IoT edge devices can be hosted in devices such as industrial controllers, MCUs, switches, routers, embedded systems or servers, and video surveillance cameras. As many such IoT devices may not have sufficient and powerful computation resources to efficiently perform AI (data science analytics) for real-time responsiveness and local interpretable and actionable data insights, it is necessary to use lightweight AI deployed and run on IoT devices (lightweight AI does not require high-end computational resources [62]). Hence, in this study, the biologically inspired RBF-ANN lightweight AI in Equation (6) is conducted over fog-cloud analytics in the described architecture. Due to its simplicity in the training process, the RBF-ANN has much shorter training time than a multilayer perceptron model [63] trained by the commonly used back-propagation algorithm [62,63]. It has the merits of (1) a simple network structure and (2) conspicuous fast and high learning and generalization performance [23,64,65].

## 3. Experiments

### 3.1. Demo Prototype and Evaluation

**Figure 7:**
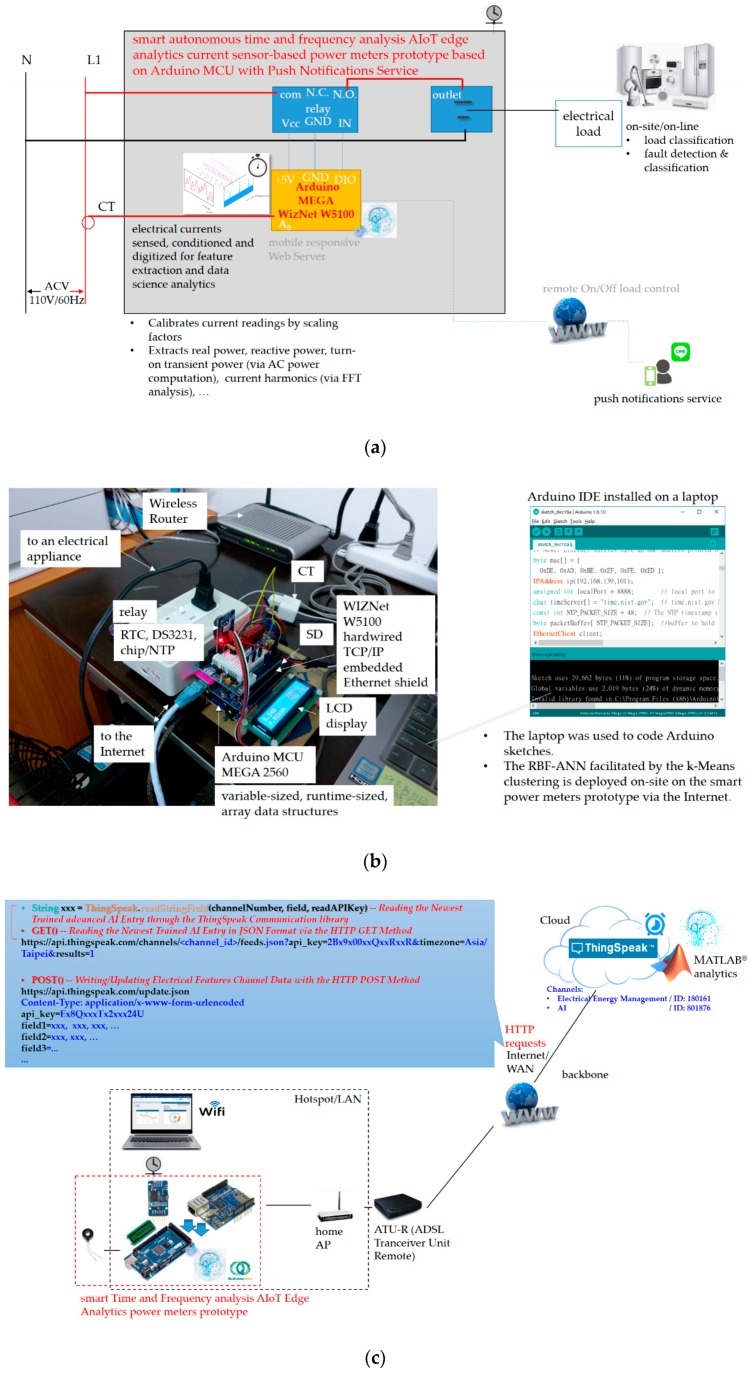
Proof-of-concept demonstration of the developed smart autonomous time and frequency analysis current sensor-based power meter prototype as edge analytics in the launched edge analytics-based AIoT architecture. (**a**) Sketch of the prototype designed, implemented, and installed for an electrical network topology and used as a smart electrical outlet for DSM in a smart grid. (**b**) Experimental setup of the prototype experimentally and practically evaluated (the prototype developed and experimentally tested in a laboratory environment in [23] is upgraded in this study). (**c**) The prototype automatically communicates with the ThingSpeak™ cloud via the Internet (updating ThingSpeak™ channel data with HTTP GET() or POST()). Its total controllability was shown in [23].

In this section, the developed smart autonomous time and frequency analysis current sensor-based power meter prototype in the edge analytics-based AIoT architecture launched with the open and powerful ThingSpeak™ IoT platform with MATLAB^®^ analytics as cloud analytics for DSM in a smart grid is experimentally and practically demonstrated. Figure 7 shows the proof-of-concept demonstration of the developed prototype. The prototype is installed for an electrical network topology and used as a smart electrical outlet (wall socket), as shown in Figure 7a. Figure 7b shows the experimental setup of the prototype where advanced AI well trained by Algorithm 1 in the ThingSpeak™ cloud is autonomously and automatically deployed via the Internet, as shown in Figure 7c.

As shown in Figure 7b, we assembled hardware and software of the Arduino MEGA 2560 MCU mounted with the Arduino W5100 Ethernet shield, which was used to: (1) acquire current signals sensed by the CT and conditioned by the MCU; (2) extract and identify electrical features extracted through the time- and frequency-domain analysis from the conditioned and digitized current signals and identified by advanced AI autonomously and automatically deployed onsite; (3) transmit data (such as electrical energy consumption and electrical features including P [46,47], turn-on transient power [46], and current harmonics) to and retrieve data (such as trained advanced AI entries) from the ThingSpeak™ cloud via the Internet (refer to Figure 7c, showing the prototype working over fog-cloud analytics); and (4) provide mobile push notification service to end users for DSM. In the fog-cloud analytics architecture in this study, load identification for DSM shown in Figure 2 and Figure 3 is performed onsite and online. The prototype can be further developed based on analysis of daily electrical energy consumption for DSM, to disaggregate whole-house load data into appliance-level load information with no intrusive deployment of individual smart plugs installed for electrical appliances monitored in a residential field of interest. 

Figure 7c shows that the prototype in Figure 7a,b communicates with the ThingSpeak™ cloud via the Internet, where they collaborate to work over fog-cloud analytics for load identification in DSM. The ThingSpeak™ cloud configured for advanced AI is shown later.

In the experiment demonstrated in this study, instantaneous current signals sensed and conditioned are acquired and the electrical features, P, turn-on transient power, and current harmonics are extracted by the prototype shown in Figure 7a,b from the acquired instantaneous current signals. According to the Nyquist–Shannon sampling theorem, in which the sampling frequency specified to indicate how frequently a signal one is trying to acquire is sampled per second needs to be at least twice the frequency of the signal acquired, the sampling frequency specified in this experiment is 2 kHz (0.5 ms/sample).

**Algorithm 1.** The two-stage method developed for the advanced AI k-means clustering-integrated RBF-ANN over fog-cloud analytics in this study.• Use k-means clustering on a training dataset collected onsite, $ℑ={\left\{X_{k}{,}^{}d_{k}\right\}}_{k=1}^{Q}$, to heuristically determine the center and spread parameters of each Gaussian basis function of the RBF-ANN of Equation (1). ↬ **Require** the values of the number of cluster centers *c* and a small constant of the tolerance *ε* to be specified. ↬ **Randomly initialize** the *c* cluster centers, where *c* has been specified. *l* ← 0 ↬ **Repeat**
    **Compute** the *c* cluster centers using Equation (8).
    **Update** the hard c-partition space *U* using Equation (9).
  *l* ← *l* + 1  **Until**
   ||*U*^(*l*+1)^ − *U*^(*l*)^|| is less than the specified small tolerance *ε*. ↬ **Return** the resulting *c* cluster centers with the hard c-partition space for ℑ. ↬ **Center** the Gaussian basis functions of Equation (1) at the returned *c* cluster centers. ↬ **Compute** the spread parameters of the centered Gaussian basis functions of Equation (1) using Equation (7). • Use Equation (6) involving the SVD technique applied to the training dataset collected onsite, ℑ, to train the RBF-ANN of Equation (1) characterized by k-means clustering. ↬ **Train** Equation (1), whose Gaussian basis functions were heuristically determined by k-means clustering applied to the training data points collected onsite using Equation (6) in the ThingSpeak^TM^ cloud to get *W*. The well-trained RBF-ANN is then autonomously and automatically deployed onsite on the developed smart autonomous power meter prototype as edge analytics via the Internet for online load identification in DSM. In this study, the RBF-ANN facilitated with k-means clustering is developed over fog-cloud analytics.

This sampling frequency means analog current signals conditioned and acquired for the time- and frequency-domain analysis are sampled 2000 times per second (i.e., sampled in a time resolution of 1/2000th of a second); the high-speed predictable interrupts enabled by the Arduino MEGA 2560 MCU are specified through the configurable submillisecond timer resolution of the FlexiTimer2 library [55]. The specified sampling frequency limits the speed at which analog current signals from monitored electrical appliances can be sampled.

**Figure 8:**
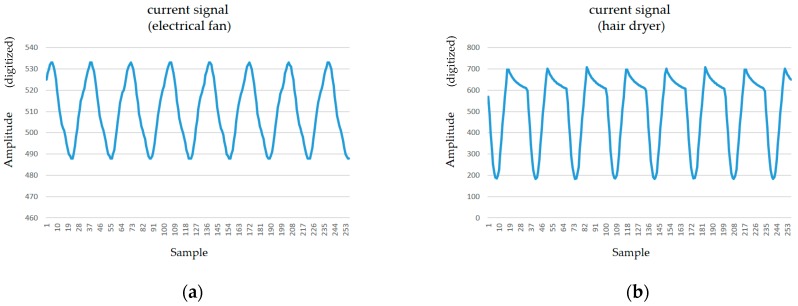
Current signals conditioned and acquired from (**a**) an electric fan and (**b**) a hair dryer.

**Figure 9:**
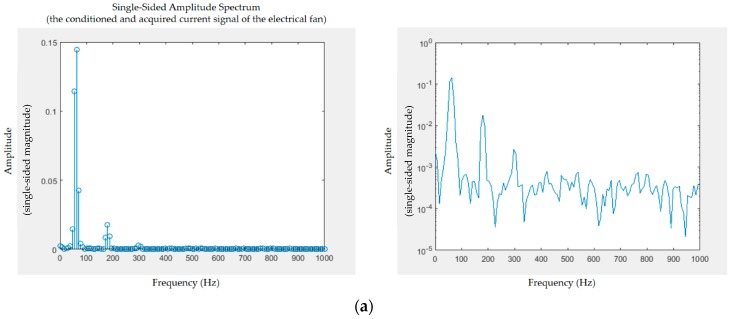

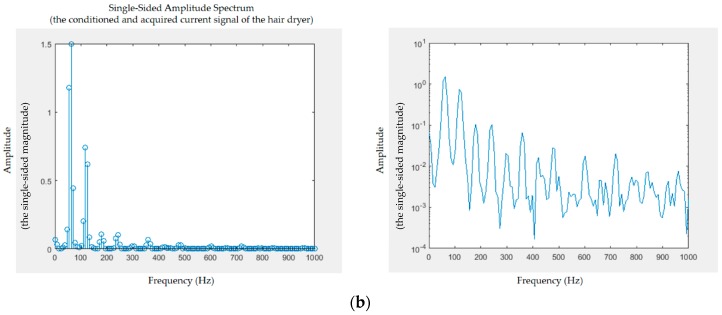
Frequency-domain single-sided amplitude spectra: (**a**) fast Fourier transform (FFT) applied for (**a**) electric fan in Figure 8a, and (**b**) hair dryer in Figure 8b. The fundamental frequency of the conditioned, acquired, and digitized current signals transformed is 60 Hz. On average, longer signals produce better frequency approximation. Note the logarithmic scale.

Figure 8 shows the analog current signals conditioned and acquired by the specified MCU’s analog pin in Figure 7a, from two electrical appliances, an electric fan (Figure 8a) and a hair dryer (Figure 8b). The conditioned and acquired analog current signals have an amplitude and offset such that they almost span the complete range of 0–5 V, well suiting the MCU’s ADC properties. Figure 9 shows the single-sided amplitude spectrums by FFT applied on the conditioned and acquired analog current signals in Figure 8. 

The conditioned and acquired analog current signals from the two electrical appliances are transformed through FFT from the time domain to the frequency domain, which shows that the current harmonics produced by the two appliances are distinguished. FFT enables seeing which frequencies are present and understanding which is the most dominant in analog signals conditioned, acquired, and digitized to be transformed, which works with a finite number of samples of such signals.

In the FFT applied in this study, the Hanning window, a mathematical function expressed as w(n)=0.5(1−cos(2πnN)),0≤n≤N (window length *L* = *N* + 1) [53], was used for general data analysis with a good trade-off between frequency and amplitude to mitigate the spectral leakage (i.e., the leakage effect) by smoothing the sinusoidal signals added together with different levels of frequency and amplitude in the time domain. Due to the 256 samples transformed through FFT in this experiment, a total of 128 bins (shown in Figure 9) distributed over 0–1 kHz because of the specified sampling frequency of 2 kHz were obtained; the number of bins obtained through FFT is half the amount of samples spanning the frequency range from 0 to half the sampling frequency. In the FFT implemented and run on the Arduino^®^ MCU, the number of samples transformed (256 in this study) must always be a power of 2, and the specified sampling frequency (2 kHz in this study) has to comply with the Nyquist–Shannon sampling theorem and be less than the maximum reading rate of *analogRead()* (10 kHz in this study) due to the technical specification of the MCU’s ADC.

Table 2 lists the current harmonics produced by the two electrical appliances and obtained through FFT in this experiment. As shown in Figure 9a,b, patterns and levels of amplitude (magnitude) at certain frequencies are used as electrical features to distinguish between the two appliances analyzed for feature extraction and used for load identification. 

**Figure 10:**
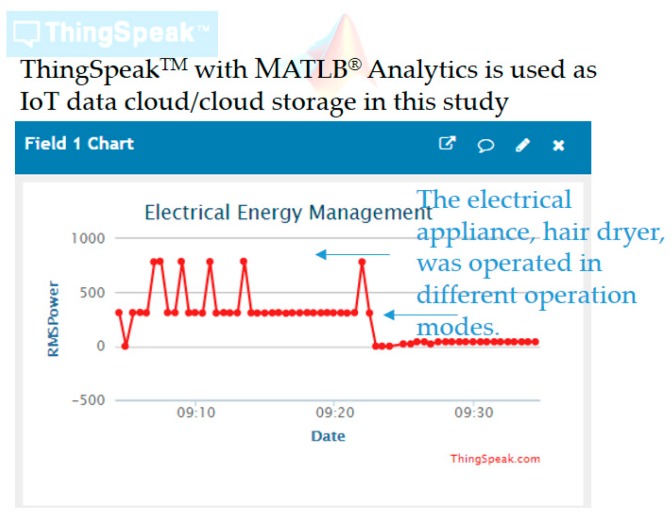
Simple web page visualizing electrical energy consumption identified by the developed smart autonomous power meter prototype in this study.

Current harmonics are needed, as ambiguous electrical features in △P and △Q (reactive power) [47] exist realistically for load identification; spectrum analysis conducted for feature extraction is suitable to complete load identification. More representative electrical features, such as total harmonic distortion of current signals computed from current harmonics, can be further extracted for feature extraction, evaluated for feature selection, and used for load identification. The open and powerful ThingSpeak™ cloud [36] configured in this study offers free cloud storage for electrical features extracted by the smart autonomous power meter prototype from different types of monitored electrical appliances. Table 3 summarizes the fields of two ThingSpeak™ channels, electrical energy management and (advanced) AI, configured over fog-cloud analytics for load identification in DSM in this study. Table 4 shows the code by which extracted electrical features are uploaded (transmitted) to the ThingSpeak™ cloud for advanced AI, which is executed by the prototype shown in Figure 3 and Figure 7. Figure 10 shows electrical energy consumption identified by the prototype, where a simple web page is provided by ThingSpeak™ to user clients for data visualization.

The ThingSpeak™ cloud used and configured in this study also offers data science analytics that provides MATLAB^®^ analytics, and collected and stored electrical features are learned by advanced AI, the k-means clustering integrated with RBF-ANN described in Section 2.2, implemented in MATLAB^®^ programming language and trained offline in cloud analytics. The k-means clustering-combined RBF-ANN is autonomously and automatically deployed via the Internet on the developed prototype for load identification (DSM) performed onsite and online.

**Figure 11:**
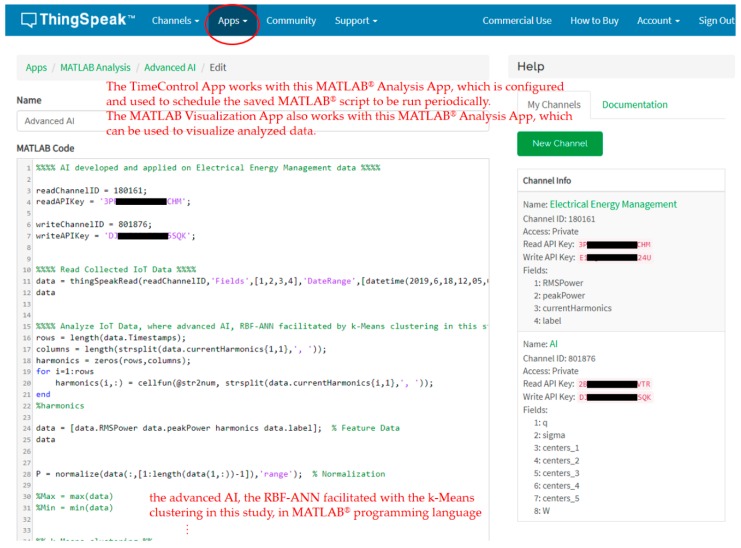
MATLAB^®^ script coded for advanced AI in this experiment.

Two electrical appliances, an electric fan and a hair dryer, were monitored in this experiment by the developed prototype. Electrical features, P, turn-on transient power, and current harmonics were extracted from the two appliances, transmitted to the ThingSpeak™ cloud for advanced AI, and identified by the prototype once the advanced AI applied on stored electrical feature data in the ThingSpeak™ cloud was well trained and autonomously and automatically deployed onsite on the prototype via the Internet for on-demand data science analytics for DSM. Figure 11 shows the MATLAB^®^ script coded for advanced AI in this study, where the following steps were applied:

(1) Use the MATLAB^®^ Analysis app [66,67] to read, analyze, and write data for advanced AI (data science analytics) implemented in MATLAB^®^ programming language and trained offline in the ThingSpeak™ cloud.
  (1.1) Read collected electrical feature data.
  (1.2) Analyze collected electrical feature data accessed from the fields of the ThingSpeak™ channel, electrical energy management, where advanced AI, the RBF-ANN facilitated with k-means clustering, is investigated, implemented, and trained on the processed electrical feature data in the ThingSpeak™ cloud.
  (1.3) Write the trained RBF-ANN facilitated with k-means clustering to the fields of the ThingSpeak™ channel, where advanced AI, the trained k-means clustering-combined RBF-ANN, is retrieved by the developed prototype via the Internet (refer to Figure 7c) and autonomously and automatically deployed onsite as edge analytics for on-demand data science analytics for DSM.
(2) Use the TimeControl app [66,67] to schedule the coded MATLAB^®^ script to regularly train advanced AI, the RBF-ANN facilitated with k-means clustering, for model updates. The advanced AI trained and scheduled in the ThingSpeak™ cloud is remodeled under a specific date range of collected (historical) electrical feature data learned with timestamps in a regular periodic manner for model updates (historical electrical feature data can be weighed in chronological order for user-centric IoT-oriented applications in DSM [8] because there are more non-working than working hours in a week, activities of daily living by occupants related to electricity can lead to more electrical energy wasted during non-working hours than consumed during working hours [68,69,70]).
(3) Use the MATLAB^®^ Visualization app [66,67] to visualize analyzed data (optional).

In this study, in the k-means clustering used to initialize the RBF-ANN, the specified number of cluster centers *c* was 5, the maximum number of iterations was 1500, and the (squared) Euclidean distance metric in which data clustered together have the least Euclidean distance was adopted. A 12-5-1 network structure of the RBF-ANN was structured, and its weighting connections, *W* in Equation (6), were trained offline in the ThingSpeak™ cloud, where the number of Gaussian basis functions of the RBF-ANN in Equation (1), *q*(= *c*), was 5. The cluster centers found through the k-means clustering process and used to allocate the center parameters of the Gaussian basis functions of the RBF-ANN in Equation (1) are shown in Table 5.

The spread parameter of the Gaussian basis functions of the RBF-ANN that were computed by Equation (7) was 0.42. The input neurons of the RBF-ANN facilitated by the k-means clustering directly convey the normalized electrical features to the neurons in the hidden layer, where its output neuron is labeled as {0 (none), 1, 2, 3,..., *N* (total number of electrical appliances monitored and identified)}, normalized, and represented as a load indicator to indicate the identified electrical appliance that is present and active.

The RBF-ANN initialized by k-means clustering was well trained by Equation (6), where λ was 0.05 in the ThingSpeak™ cloud. In this experiment, the collected training dataset, electrical features used as input variables of the RBF-ANN, was normalized so that its range is in the interval [0, 1]. Table 6 shows the well-trained RBF-ANN, *W*, in this study.

The well-trained and updated advanced AI in Table 5 and Table 6 was autonomously and automatically deployed onsite on (ported on) the developed prototype via the Internet [71,72], where: (1) present time was retrieved from the RTC chip and recognized by the prototype for autonomous AI updates, and (2) the ThingSpeak Communication library [72] was included in the programmed Arduino sketch in Figure 7b and used to read the newest trained advanced AI entry from the fields of the created AI channel in the ThingSpeak™ cloud for the latest advanced AI update. The ThingSpeak Communication library enables an Arduino MCU or compatible hardware to read data from or write data to the ThingSpeak™ cloud. The latest advanced AI update was autonomously and automatically deployed onsite on (ported on) the prototype via the Internet as edge analytics for DSM in this study (refer to Figure 7c). ArduinoJson [73], a C++ JavaScript^®^ Object Notation (JSON) [74,75] library for Arduino^®^ and IoT, instead of the ThingSpeak Communication library, can be included in the programmed Arduino sketch and used to parse the responded latest JSON-formatted advanced AI.

Finally, the well-trained RBF-ANN facilitated with k-means clustering (shown in Table 5 and Table 6) was autonomously and automatically deployed onsite and run on the Arduino MEGA 2560 MCU as edge analytics for online load identification in DSM, where the programmed Arduino sketch used 43,744 bytes (17%) of the maximum program storage space of 253,952 bytes; the global variables used 5622 bytes (68%) of the maximum dynamic memory of 8192 bytes, leaving 2570 bytes for local variables. Load identification in DSM in this study was performed by the developed prototype onsite and online. Table 7 shows the overall load identification rate obtained in this study: 94.26% (a total of 122 data instances are identified, and seven data instances are misidentified). An analysis of the k-means clustering (k = 5) combined with RBF-ANN with no regularization risk functional (λ = 0) in Equation (6) was conducted and compared with k-means clustering (k = 5) combined with RBF-ANN with a regularization risk functional (λ = 0.05) in Equation (6), as shown in Table 7; the overall load identification rate was improved by 5.74%.

**Figure 12:**
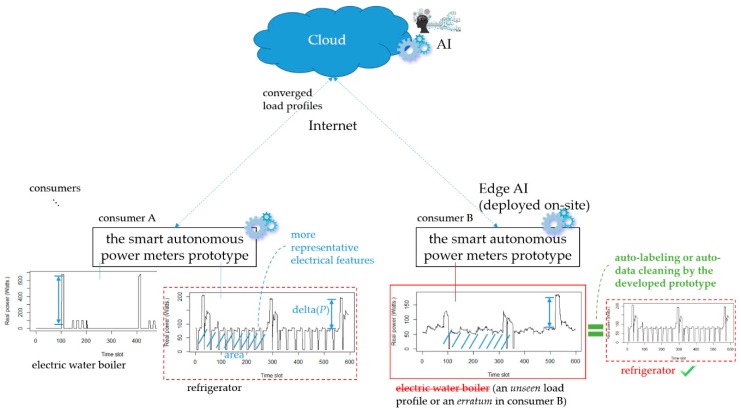
Demonstration of auto-labeling or auto-data cleaning by means of online load identification performed onsite on the developed prototype through converged analytics (as edge-to-edge connectivity).

Figure 12 demonstrates auto-labeling or auto-data cleaning by means of online load identification performed onsite on the developed prototype through converged analytics (more IoT service-oriented applications for DSM could build upon this demonstration; also refer to Figure 2). In Figure 12, two more different types of electrical home appliances, a refrigerator and an electric water boiler, can be monitored by the developed prototype considering more representative extracted electrical features, where an unseen load profile by a new appliance monitored in a practical field (consumer B) can be identified through converged analytics (leveraging consumer A to provide data insights for consumer B) by the developed prototype as edge analytics (edge AI).

**Figure 13:**
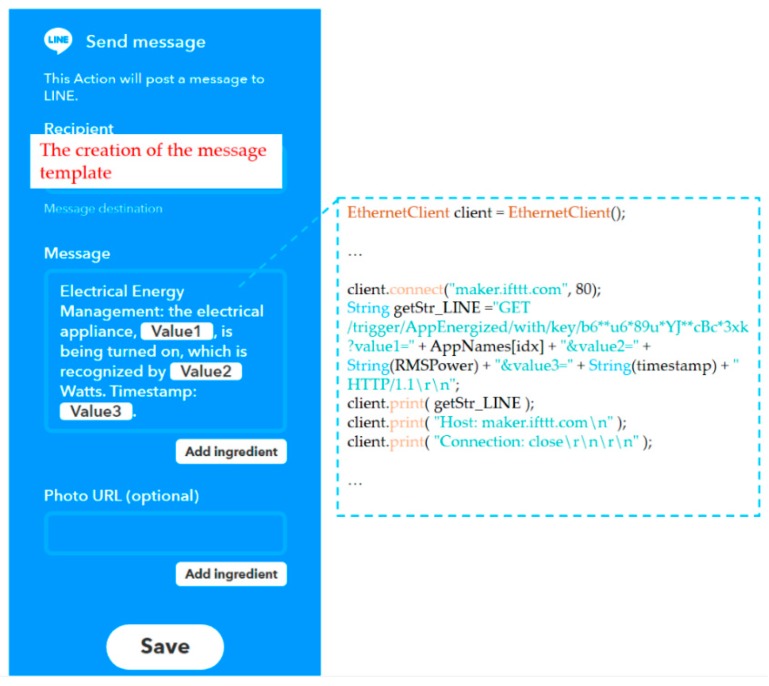
Illustration of the creation of an If-This-Then-That (IFTTT) rule with Webhooks in this experiment.

**Figure 14:**
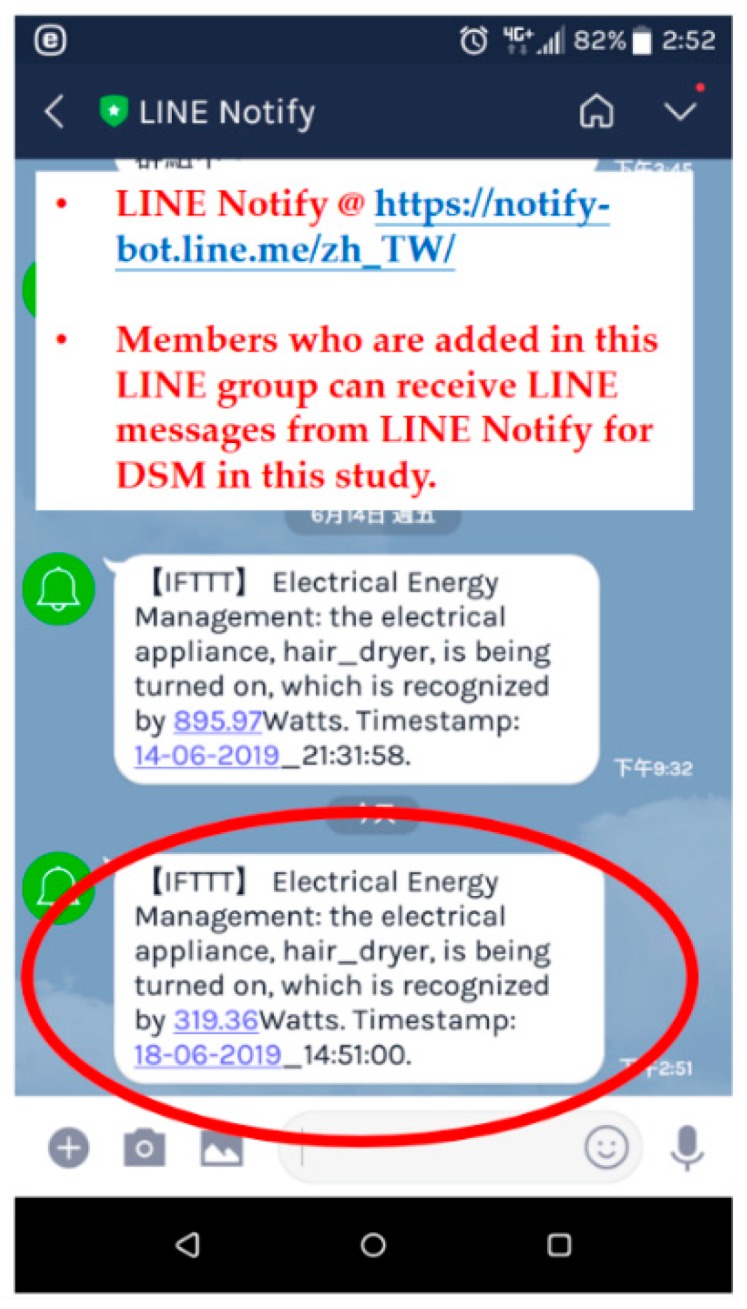
Load event message received by a LINE-Notify mobile phone over the IFTTT paradigm with Webhooks. A prespecified appliance event was identified and triggered for the monitored hair dryer.

Regarding push notification service in the fog-cloud analytics architecture in this study, a practical paradigm of IFTTT [37] with Webhooks [38] was conducted, where an IFTTT rule with Webhooks publishing a new trigger received or an action made was created for LINE-Notify mobile devices, as illustrated in Figure 13. Figure 14 shows a load event message received by a LINE-Notify mobile phone over the IFTTT paradigm with Webhooks for load identification in DSM in this study. As shown in Figure 13, the sent LINE message was received when an appliance event, the monitored hair dryer energized and identified in this experiment, identified by the developed prototype with well-trained advanced AI was triggered and published by Webhooks [38].

### 3.2. Discussion

**Figure 15:**
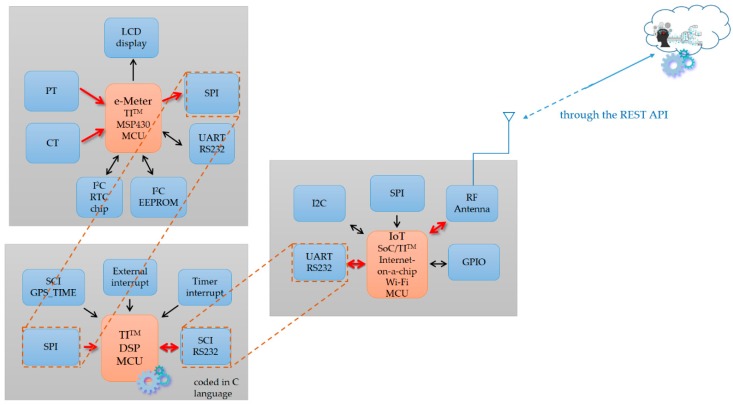
DSP MCU-versioned powerful smart autonomous power meter prototype.

Figure 7 shows the proof-of-concept demonstration of the developed smart autonomous time- and frequency-domain analysis current sensor-based power meter prototype as edge analytics in an edge analytics-based AIoT architecture launched with an open and powerful cloud analytics platform. The prototype is based on an Arduino MEGA 2560 MCU mounted with a WIZNet W5100 hardwired TCP/IP embedded Ethernet shield; the configured cloud analytics platform is based on ThingSpeak™ with MATLAB^®^ analytics. Also, the k-means clustering combined with RBF-ANN, advanced AI, is applied on electrical features, P, turn-on transient power, and current harmonics, where it is trained offline in the ThingSpeak™ cloud and autonomously and automatically deployed onsite on the developed prototype. Table 7 shows the overall load identification rate of 94.26% achieved and improved by 5.74% in that the regularization risk functional (λ = 0.05) is considered by k-means clustering combined with RBF-ANN (k = 5). Different types of electrical home appliances can be monitored by the developed prototype, as seen in Figure 2 and Figure 12. More IoT service-oriented applications for DSM in a smart grid could be based on it. A digital signal processing (DSP) MCU-versioned powerful smart autonomous power meter prototype can be conducted alternatively and developed with the functionalities of the developed prototype in the described architecture over fog-cloud analytics in this study, which is given in Figure 15. In Figure 15, a Texas Instruments (TI)™ DSP MCU integrates (1) a TI™ MSP430 MCU as an e-meter for current and voltage measurements with (2) an IoT SoC/TI^TM^ Internet-on-a-chip Wi-Fi MCU as a portfolio of cloud integration with Internet connectivity (the REST API).

## 4. Conclusions and Future Work

A smart grid is an innovative electrical energy network with two-way communication that can improve the reliability and flexibility of conventional power grids based on DSM. Fog-cloud analytics is extremely important to reduce network latency, conserve network bandwidth (throughput), and enable innovative AIoT applications. DSM, an essential part of a smart grid, enhances the efficiency, reliability, and flexibility of a traditional power grid upgraded to meet continuously increasing electrical energy demands by consumers. In this study, we designed and implemented a smart autonomous time- and frequency-domain analysis current sensor-based power meter prototype as edge analytics in an edge analytics-based AIoT architecture launched with an open and powerful cloud analytics platform for DSM in a smart grid. The prototype is based on an Arduino MEGA 2560 MCU mounted with a WIZNet W5100 hardwired TCP/IP embedded Ethernet shield; the cloud analytics platform is based on ThingSpeak™ with MATLAB^®^ analytics. Also, advanced AI, the k-means clustering combined with RBF-ANN presented in this study, is: (1) applied on electrical features, P, turn-on transient power, and current harmonics; (2) trained offline in the ThingSpeak™ cloud; and (3) autonomously and automatically deployed onsite on the developed prototype. As demonstrated and reported in this study, an overall load identification rate of 94.26%, where the regularization risk functional is considered, is achieved for auto-labeling or auto-data cleaning by means of online load identification performed onsite on the developed prototype through converged analytics. More IoT service-oriented applications for DSM could build upon it. The prototype designed and implemented to collaborate with the configured ThingSpeak™ cloud over fog-cloud analytics for DSM in this study is feasible and workable.

An ensemble-based AI model such as multi-label classification based on random forest algorithms [76] will be developed and evaluated in the future, as various AI algorithms are effective approaches that can be used for load classification and long short-term load prediction in electrical energy management [77,78]. In the future, the developed smart autonomous power meter prototype in this study will be investigated for prognostics and health management in industry 4.0, where the availability and efficiency of equipment monitored by the prototype further investigated will be assessed. Electric energy consumption data from all consumers accumulate as Big Data (which is very computationally intense) in a power utility’s cloud server, which will also be investigated and addressed through modern graphics processing unit technology.

## Figures and Tables

**Table 1:** Technical specifications of Arduino MEGA 2560 micro-controller unit (MCU) [21,23,39]. SRAM, static random access memory; EEPROM, electrically erasable programmable read-only memory; PWM, pulse width modulation.

Arduino^®^ MCU board	ATmega 2560
Operating voltage	5 V
Input voltage (recommended)	7–12 V
Digital input/output (I/O) pins	54 (of which 15 provide PWM output)
Analog input pins	16
Direct current (DC) per I/O pin	20 mA
DC for 3.3 V pin	50 mA
Flash memory	256 KB (8 KB used by the bootloader)
SRAM	8 KB
EEPROM	4 KB
Clock speed	16 MHz
LED_BUILTIN, number of pins on-board LED is connected to	13
Size (length × width)	101.52 mm × 53.3 mm

**Table 2:** Resulting current harmonics produced by two types of electrical appliances and obtained through fast Fourier transform (FFT) in this experiment.

Electric Fan	**DC Offset**	**Fundamental Harmonic (60 Hz)**	**2nd-Order Harmonic**	**3rd-Order Harmonic**	**4th-Order Harmonic**	**5th-Order Harmonic**
	0.002	0.144	0.000	0.018	0.000	0.003
	**6th-order harmonic**	**7th-order harmonic**	**8th-order harmonic**	**9th-order harmonic**		
	0.000	0.001	0.001	0.001		
Hair Dryer	**DC Offset**	**Fundamental Harmonic (60 Hz)**	**2nd-Order Harmonic**	**3rd-Order Harmonic**	**4th-Order Harmonic**	**5th-Order Harmonic**
	0.070	1.498	0.741	0.105	0.103	0.020
	**6th-Order Harmonic**	**7th-Order Harmonic**	**8th-Order Harmonic**	**9th-Order Harmonic**		
	0.066	0.016	0.028	0.002		

**Table 3:** Fields of two ThingSpeak™ channels configured over fog-cloud analytics for load identification in DSM in this study.

Channel	Field	Physical Meaning/Sensor
Electrical energy management	Field 1 (RMSPower ^1^)	Real power/CT
	Field 2 (Ptransient/peakPower)	Turn-on transient power/CT
	Field 3 (maximum magnitude up to 9th-order current harmonics)	Current harmonics/CT
	Field 4 (label)	Advanced AI
AI (RBF-ANN facilitated by k-means clustering)	Field 1 (*q* (= *c* by k-means clustering))	–
	Field 2 (σ)	–
	Field 3 (centers by k-means clustering)	–
	Field 4 (centers by k-means clustering)	–
	Field 5 (centers by k-means clustering)	–
	Field 6 (centers by k-means clustering)	–
	Field 7 (centers by k-means clustering)	–
	Field 8 (*W*)	–

^1^ Real power computed at each time interval, for example, 1 s.

**Table 4:** Code executed by the developed smart autonomous power meter prototype and used to upload (transmit) extracted electrical features to the ThingSpeak™ cloud for data storage to be further analyzed in cloud analytics.

/* … */ … String currentHarmonics = ““; byte server[] = { 184, 106, 153, 149 }; // IP address (or api.thingspeak.com) for ThingSpeak (https://thingspeak.com/) String writeAPIKey = “Ex8QxxxTxxxxxx4U”; // Write API key for a ThingSpeak channel … … updateThingSpeak(“field1=“ + String(RMSPower) + “&field2=“ + String(peakPower) + “&field3=“ + currentHarmonics); … … void updateThingSpeak(String tsData) { // RMSPower, peakPower (Ptransient), current harmonics, …more if (client.connect(server, 80)) { Serial.println(F(“Connected to ThingSpeak...”)); client.print(“POST /update HTTP/1.1\n”); client.print(“Host: api.thingspeak.com\n”); client.print(“Connection: close\n”); client.print(“X-THINGSPEAKAPIKEY: “+writeAPIKey+”\n”); client.print(“Content-Type: application/x-www-form-urlencoded\n”); client.print(“Content-Length: “); client.print(tsData.length()); client.print(“\n\n”); client.print(tsData); } … } …

**Table 5:** Center parameters found through k-means clustering are used to characterize the RBF-ANN of Equation (1), where electrical feature data are normalized in this experiment.

Cluster center 1	**P**	**Turn-on transient power**	**DC offset**	**Fundamental harmonic (60 Hz)**	**2nd-order harmonic**	**3rd-order harmonic**
	0.394	0.387	0.344	0.442	0.985	0.883
	**4th-order harmonic**	**5th-order harmonic**	**6th-order harmonic**	**7th-order harmonic**	**8th-order harmonic**	**9th order harmonic**
	0.903	0.676	0.865	0.529	0.829	0
Cluster center 2	**P**	**Turn-on transient power**	**DC offset**	**Fundamental harmonic (60 Hz)**	**2nd-order harmonic**	**3rd-order harmonic**
	0.997	0.984	0.684	0.990	0.022	0.152
	**4th-order harmonic**	**5th-order harmonic**	**6th-order harmonic**	**7th-order harmonic**	**8th-order harmonic**	**9th-order harmonic**
	0.067	0.611	0.024	1.000	0.056	0.333
Cluster center 3	**P**	**Turn-on transient power**	**DC offset**	**Fundamental harmonic (60 Hz)**	**2nd-order harmonic**	**3rd-order harmonic**
	0.048	0.050	0.071	0.059	0	0.168
	**4th-order harmonic**	**5th-order harmonic**	**6th-order harmonic**	**7th-order harmonic**	**8th-order harmonic**	**9th-order harmonic**
	0	0	0	0	0	0
Cluster center 4	**P**	**Turn-on transient power**	**DC offset**	**Fundamental harmonic (60 Hz)**	**2nd-order harmonic**	**3rd-order harmonic**
	0.391	1.000	0.474	0.437	0.970	0.818
	**4th-order harmonic**	**5th-order harmonic**	**6th-order harmonic**	**7th-order harmonic**	**8th-order harmonic**	**9th-order harmonic**
	0.900	0.667	0.857	0.500	0.667	0
Cluster center 5	**P**	**Turn-on transient power**	**DC offset**	**Fundamental harmonic (60-Hz)**	**2nd-order harmonic**	**3rd-order harmonic**
	0	0	0.042	0	0	0
	**4th-order harmonic**	**5th-order harmonic**	**6th-order harmonic**	**7th-order harmonic**	**8th-order harmonic**	**9th-order harmonic**
	0	0	0	0	0	0

**Table 6:** *W*.

*W*	0.478
	0.901
	4.966
	0.320
	−4.445

**Table 7:** Overall load identification rate achieved in this study.

	K-means clustering (k = 5) combined with RBF-ANN (λ = 0.05) well trained in the cloud is autonomously and automatically deployed as edge analytics on the developed prototype for onsite and online load identification in DSM	K-means clustering (k = 5) combined with RBF-ANN (λ = 0)	Overall load identification rate improvement (%)
Overall load identification rate (%)	94.26	88.52	+5.74
